# Integrase Strand Transfer Inhibitors for Treatment-experienced Young Adults With Perinatal HIV in the US: Immunologic, Virologic, and Anthropometric Outcomes

**DOI:** 10.1093/cid/ciaf538

**Published:** 2026-02-09

**Authors:** Kunjal Patel, Alicia Jaramillo-Underwood, Brad Karalius, Tzy-Jyun Yao, Russell B. Van Dyke, Ayesha Mirza, Jennifer Jao, Allison L. Agwu

**Affiliations:** 1 Department of Epidemiology, Harvard T.H. Chan School of Public Health, Boston, Massachusetts, USA; 2 Center for Biostatistics in AIDS Research, Harvard T.H. Chan School of Public Health, Boston, Massachusetts, USA; 3 Department of Pediatrics, Tulane University School of Medicine, New Orleans, Louisiana, USA; 4 Department of Pediatrics, University of Florida College of Medicine, Jacksonville, Florida, USA; 5 Department of Pediatrics, Northwestern University Feinberg School of Medicine, Chicago, Illinois, USA; 6 Division of Pediatric Infectious Diseases, Department of Pediatrics, Johns Hopkins School of Medicine, Baltimore, Maryland, USA; 7 Division of Infectious Diseases, Department of Medicine, Johns Hopkins School of Medicine, Baltimore, Maryland, USA

**Keywords:** integrase strand transfer inhibitors, perinatal HIV, HIV viral load, CD4 count, weight gain

## Abstract

**Background.:**

The efficacy of integrase strand transfer inhibitors (INSTIs) among adults with HIV is well-established, though adverse effects, particularly weight gain, are common. Comparable data for treatment-experienced adolescents and young adults with perinatally-acquired HIV (AYAPHIV) are limited.

**Methods.:**

AYAPHIV in the US-based Pediatric HIV/AIDS Cohort Study who switched to bictegravir (BIC), dolutegravir (DTG), elvitegravir (EVG), or raltegravir (RAL) from any prior regimen were eligible. Using mixed-effects models, viral load, CD4 count, weight, and body mass index were described through 2 years after switch for each INSTI.

**Results.:**

Among 556 AYAPHIV, there were 167 switches to BIC, 282 to DTG, 189 to EVG, and 151 to RAL. Viral suppression (<200 copies/mL) at 1 and 2 years after switch was 74% and 69% for BIC, 62% and 60% for DTG, 76% and 68% for EVG, and 58% and 52% for RAL. Mean CD4 counts were above 500 cells/mm^3^ after switch through 2 years for all INSTIs. Average weight gain in the first year after switch to BIC, DTG, EVG, and RAL was 0.2, 2.5, 3.8, and −0.2 kilograms for females and 2.3, 4.8, 2.9, and 2.6 kilograms for males. Among previously underweight/healthy individuals, 13%, 18%, 36%, and 12% of females and 6%, 8%, 12%, and 11% of males switching to BIC, DTG, EVG, and RAL were overweight/obese by 2 years after switch.

**Conclusions.:**

Individual INSTI-based regimens among treatment-experienced AYAPHIV had moderate effectiveness with respect to viral suppression. Continued average weight gain across INSTIs raises concerns about long-term cardiometabolic sequalae.

Among treatment-naive adults with HIV-1, integrase strand transfer inhibitor (INSTI)-based regimens including bictegravir (BIC), dolutegravir (DTG), elvitegravir (EVG), and raltegravir (RAL) have been shown to be effective, tolerable, have a high barrier to resistance, and have few drug interactions [1-6]. Studies of these regimens among antiretroviral treatment (ART)-experienced adults are heterogeneous between specific INSTIs but cumulatively suggest a benefit of switching to INSTIs, with higher effectiveness among virologically suppressed versus viremic adults at the time of switch [7-17]. While these studies help inform treatment for older adolescents and young adults with perinatally acquired HIV (AYAPHIV), they primarily include mature adults who acquired HIV later in life and none have reported outcomes separately for this unique population with HIV ageing into adulthood.

Although AYAPHIV share some similarities with ART-experienced adults, their life long exposure to HIV through gestation, early infancy, childhood, and puberty makes them distinct in ways that may impact response to different HIV treatment strategies [18]. Additionally, given the ART era in which they were born and the often-prolonged delays in approving new, more effective treatments in children, ART-experienced AYAPHIV often have complex resistance profiles, which may differ from ART-experienced adults because of archived resistance over a lifetime of exposure to ART and potential non-adherence [19, 20]. They also differ significantly from adults with regard to age-related differences in psychosocial stressors, and immunologic, metabolic, functional, and comorbidity profiles at the time of initiation of particular ART regimens [18]. Data are, therefore, needed that specifically evaluate effectiveness of INSTI-based regimens among treatment-experienced AYAPHIV.

Further, given reports about weight gain with INSTI use among adults [21-26], there is a need to evaluate weight changes specifically among AYAPHIV. There is mounting evidence of high rates of obesity-related early comorbidities including hypertension and insulin resistance in this population [27, 28]. Additionally, overweight and obesity in adolescence and young adulthood are often associated with depression, anxiety, social stigma, and other psychosocial consequences [29]. The few studies that evaluated weight gain among youth with HIV focused on younger children and adolescents initiating INSTIs, predominantly DTG [30-36]. One study focused on 66 older AYAPHIV in Italy and did not observe excessive weight gain with switching to an INSTI-based regimen [37].

We provide comprehensive data on HIV plasma viral load, CD4 count, weight, body mass index (BMI), and treatment discontinuation at 1 and 2 years after switch to BIC-, DTG-, EVG, and RAL-based regimens in a large, diverse cohort of AYAPHIV in the United States.

## METHODS

### Study Population

The Pediatric HIV/AIDS Cohort Study Adolescent Master Protocol (AMP) and AMP Up studies were designed to evaluate the long-term health effects of PHIV and ART among children, adolescents, and young adults. In March 2007, 15 sites in the US including Puerto Rico began enrolling children and adolescents between 7 and <16 years of age with PHIV and a comparison cohort exposed to but without HIV in AMP with follow-up ending in February 2020. In May 2014, 14 sites began enrolling young adults 18 years of age and older with and without PHIV into AMP Up, including those who aged up from AMP, with follow-up ending in March 2025. Both the AMP and AMP Up protocols were approved by the Institutional Review Board at each participating site and the Harvard T.H. Chan School of Public Health. Written informed consent was obtained from each participant or their authorized representative.

We evaluated participants with PHIV enrolled in the AMP and AMP Up studies through 1 October 2023, who had a recorded treatment switch to a regimen including BIC, DTG, EVG, and/or RAL.

### Primary Exposures and Outcomes

ART history was abstracted from medical charts. Lifetime treatment history was used to identify the first recorded switch to BIC, DTG, EVG, and/or RAL for participants in AMP and treatment history from entry onwards was used to identify the first study-recorded switch to BIC, DTG, EVG, and/or RAL for participants in AMP Up who were not previously enrolled in AMP. Participants who switched from 1 INSTI to another (eg, RAL to DTG) were allowed to contribute to outcomes for each INSTI they received from the time of switch to either discontinuation or end of follow-up. Outcomes of interest included time to treatment discontinuation as well as viral suppression (<200 copies/mL), CD4 count (cells/mm^3^), and BMI (kg/m^2^) at the time of first switch to a particular INSTI and at 1 and 2 years after switch. BMI was categorized as underweight (<18.5 kg/m^2^), healthy weight (18.5 to <25.0 kg/m^2^), overweight (25.0 to <30.0 kg/m^2^), or obese (≥30.0 kg/m^2^). Additional outcomes included changes in weight (kg) and BMI from 1 year prior to first recorded switch to 1 year after switch to a particular INSTI.

### Statistical Methods

Characteristics at time of first recorded switch to BIC, DTG, EVG, and/or RAL and at time of INSTI discontinuation (if observed) were summarized for all participants who had a recorded switch. Kaplan–Meier methods were used to estimate median time to discontinuation of each INSTI.

Given that age at regimen switch varied widely between INSTIs, all further analyses were restricted to participants who switched to an INSTI between 15 and 29 years of age to allow for comparability. Follow-up periods during which participants experienced a pregnancy in the year prior to switch to a particular INSTI or during follow-up after switch were also excluded. Piece-wise linear mixed effects models with random intercepts and slopes were used to estimate HIV plasma viral loads, CD4 counts, weight, and BMI during the year prior to switch and through 2 years after switch for each INSTI. Participants were required to have at least 2 outcome measurements with at least one after switch to a particular INSTI to be included. We a priori chose 3 times at which slopes were allowed to change (ie, knots): at 1 year before switch, at time of switch, and at 1 year of follow-up after switch to a particular INSTI. We applied a nested correlation structure to account for participants who contributed more than 1 longitudinal series of data points by having been prescribed different INSTIs throughout their study follow-up. The most flexible covariance structure that resulted in model convergence was chosen. Mixed effects models for weight and BMI were adjusted for age and CD4 count at time of switch to a particular INSTI using inverse probability weighting to further account for differences between INSTIs in age at time of switch and disease severity at time of switch (ie, potential for a return to health in the setting of more advanced HIV). Ninety-five percent percentile-based bootstrap confidence intervals (CIs) with a minimum of 1000 bootstrap samples were reported for estimated slopes. Analyses were conducted using SAS version 9.4.

## RESULTS

Among 806 AYAPHIV enrolled in the AMP and AMP Up studies through 1 October 2023, 556 (69%) had at least 1 recorded treatment switch to an INSTI-based regimen (Table 1). Sixty-two percent of the study participants were female sex at birth; 61% self-reported as Black non-Hispanic, and 29% as Hispanic (regardless of race). There were 167 regimen switches to BIC, 282 to DTG, 189 to EVG, and 151 to RAL. As the newest INSTI, treatment switches to BIC occurred at older ages (median: 24 years) and were more likely to be from prior INSTI-based regimens. Median viral load at time of switch to RAL and median CD4 count at time of switch to EVG were higher than those at time of switch to the other INSTIs. A higher proportion of switches to DTG occurred at a CDC class C clinical stage (33%) compared to the other INSTIs (26–30%).

**Figure 1.**
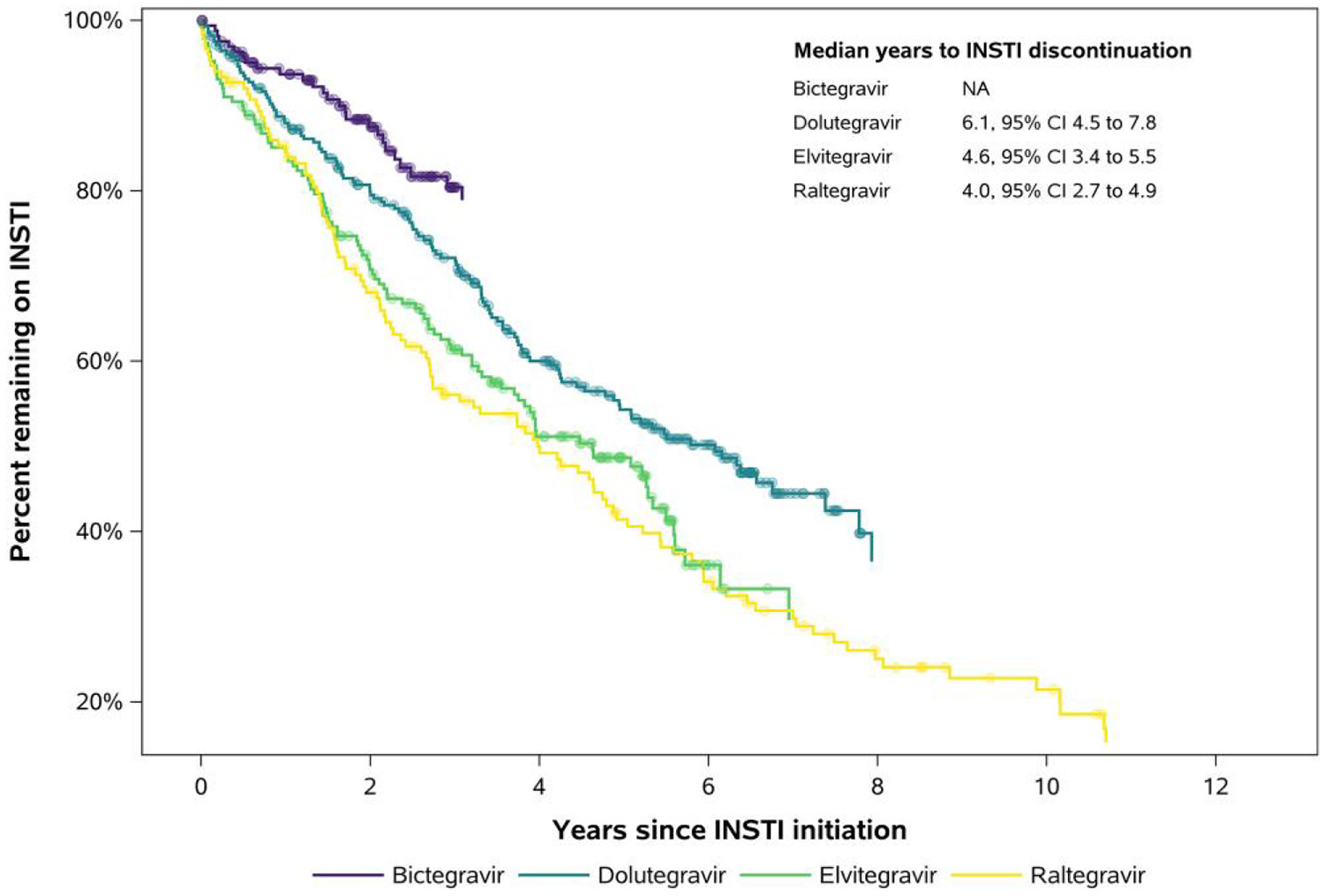
Kaplan–Meier survival curves for time to regimen discontinuation. Note: Censoring events are expressed as closed circles overlaid atop Kaplan-Meier curves.

Over follow-up, the median observed duration of time on the INSTI regimen was 2.4 years for BIC, 3.6 years for DTG, 3.2 years for EVG, and 3.1 years for RAL. We observed 26/167 (16%) discontinuations of BIC, 127/282 (45%) discontinuations of DTG, 99/189 (52%) discontinuations of EVG, and 109/151 (72%) discontinuations of RAL. Characteristics at the time of INSTI discontinuation are presented in Table 2. Median years to discontinuation were 6.1, 4.6, and 4.0 years for DTG, EVG, and RAL, respectively (Figure 1); this could not be calculated for BIC due to limited follow-up on this most recently approved INSTI.

**Figure 2.**
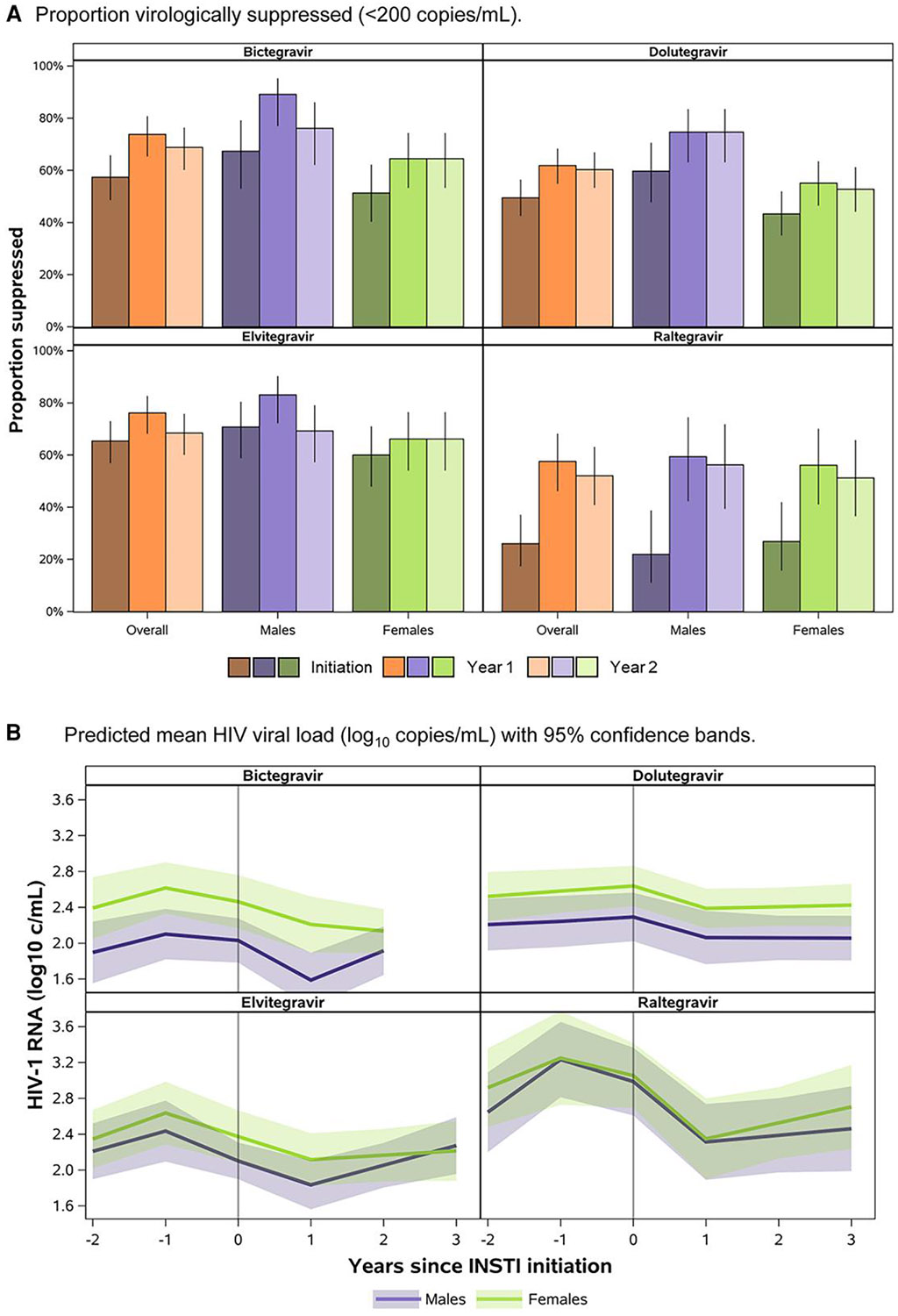
Predicted HIV viral loads through 2 y after switch to INSTI-based regimen, by sex at birth.

**Figure 3.**
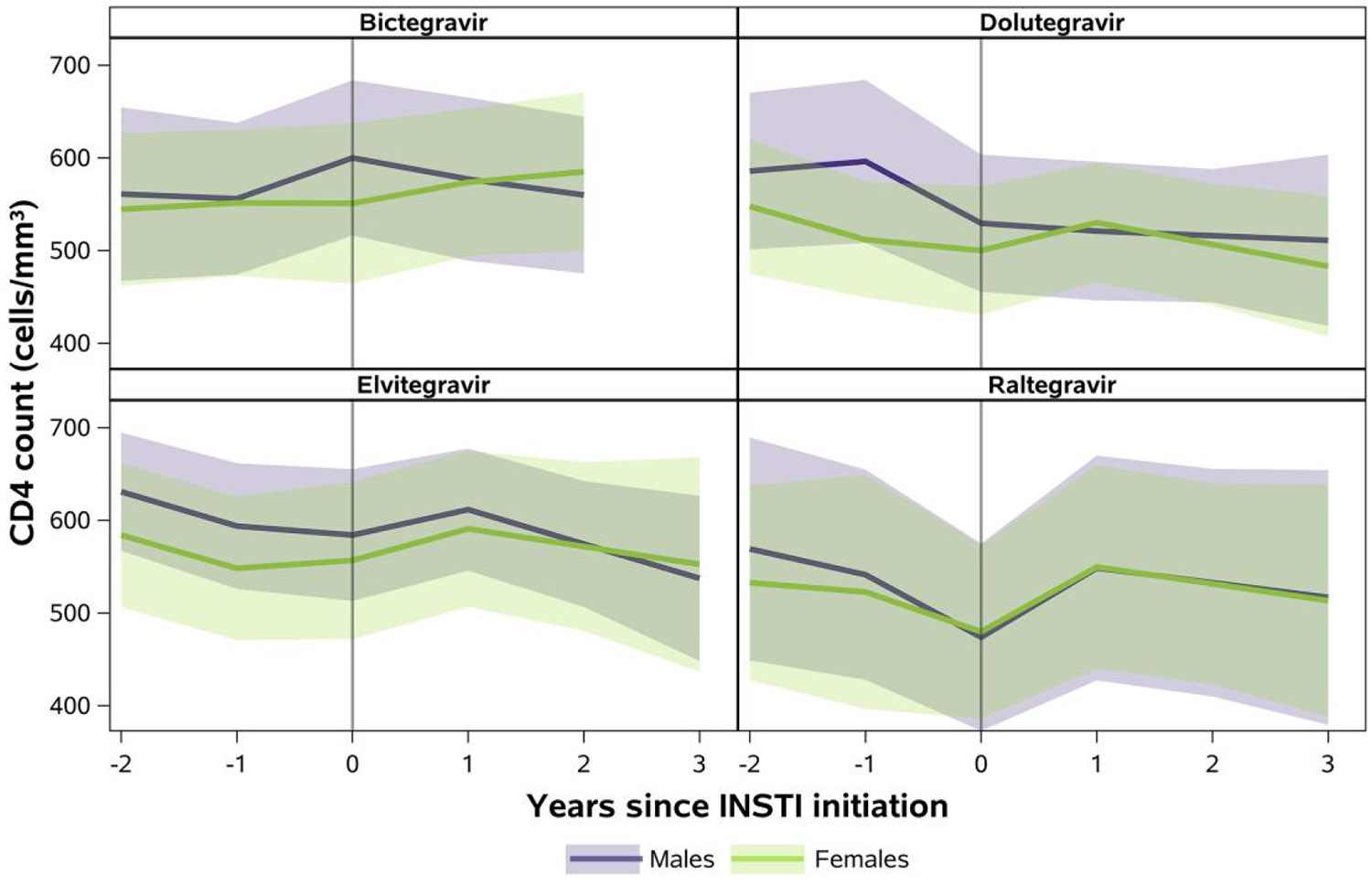
Predicted mean CD4 count (cells/mm^3^) with confidence bands through 2 y after switch to INSTI-based regimen, by sex at birth.

Mixed-effects model–based results showed fewer participants to be virally suppressed at the time of switch to RAL (26%) relative to BIC (57%), DTG (49%), and EVG (65%) (Figure 2A). Subsequently, there was a larger average decrease in HIV viral load in the first year after switch to RAL (−0.7 log_10_ copies/mL, 95% confidence interval [CI]: −1.0, −.4) relative to the other INSTIs (Figure 2B). Viral suppression at 1 and 2 years after switch was 74% and 69% for BIC, 62% and 60% for DTG, 76% and 68% for EVG, and 58% and 52% for RAL. Fewer females than males were virally suppressed from time of switch to BIC, DTG, or EVG through 2 years after switch. However, average CD4 counts were generally maintained above 500 cells/mm^3^ after switch through 2 years after switch for both females and males across all INSTIs (Figure 3).

**Figure 4.**
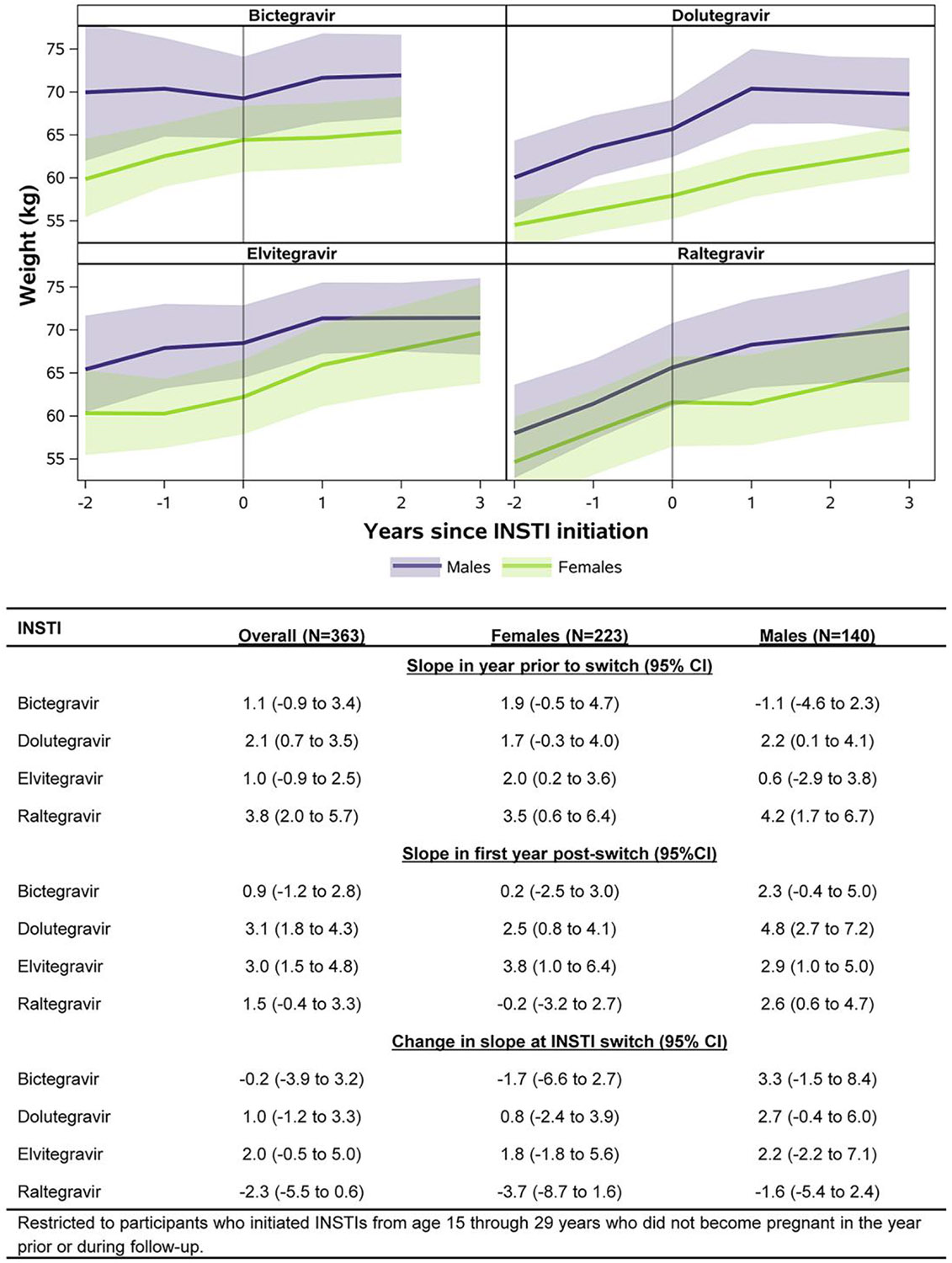
Predicted mean weight (kg) with confidence bands through 2 y after switch to INSTI-based regimen, by sex at birth.

Mixed-effects model–based results for weight showed increases among females in the first year after switch to DTG (2.5 kg, 95% CI: 0.8, 4.1) or EVG (3.8 kg, 95% CI: 1.0, 6.4), though these trajectories did not differ substantially from weight increases in the year prior to switch to these regimens (DTG: 1.7 kg, 95% CI: −0.3, 4.0; EVG: 2.0 kg, 95% CI: 0.2, 3.6; Figure 4). Similarly, among males, there were increases in weight in the first year after switch to BIC, DTG, EVG, or RAL, though these trajectories did not differ substantially from those in the year prior to switch to these regimens.

**Figure 5.**
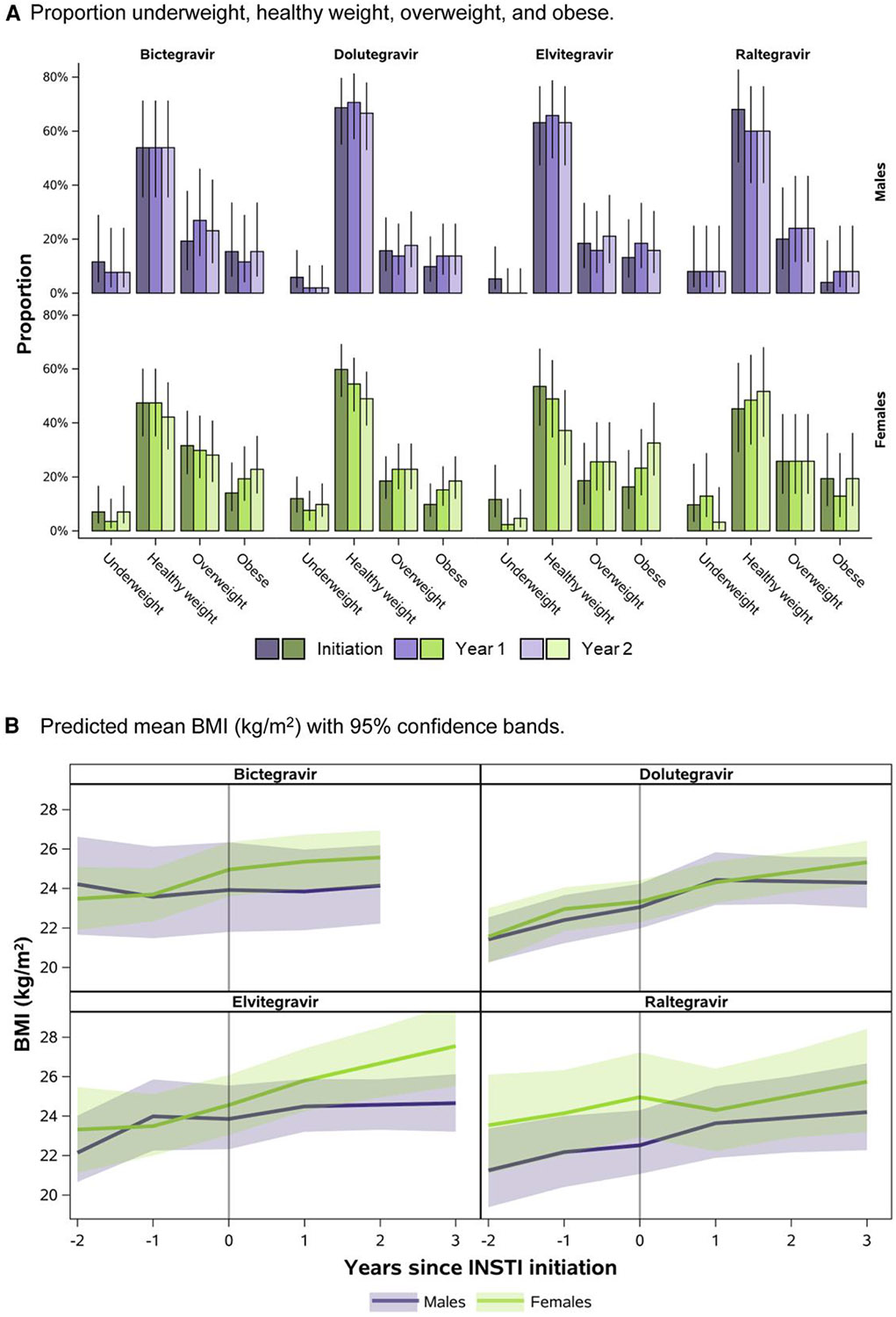
Predicted body mass index (BMI) through 2 y after switch to INSTI-based regimen, by sex at birth.

Based on mixed-effects models for BMI, 46%, 28%, 35%, and 45% of females were overweight/obese at the time of switch to BIC, DTG, EVG, or RAL, respectively (Figure 5A). In comparison, 35%, 25%, 32%, and 24% of males were overweight/obese at the time of switch to BIC, DTG, EVG, or RAL, respectively. Figure 5B shows the trajectories of BMI in the 2 years prior to switch through 2 years after switch for each INSTI, by sex at birth. Similar to results for weight, there were increases in BMI in the first year after switch to DTG (1.0 kg/m^2^, 95% CI: 0.4, 1.6) and EVG (1.3 kg/m^2^, 95% CI: 0.3, 2.4) among females, though these slopes were similar to those prior to switch to these INSTIs. Among males, there were increases in BMI in the first year after switch to DTG (1.4 kg/m^2^, 95% CI: 0.7, 2.2) and RAL (1.0 kg/m^2^, 95% CI: −0.1, 1.9), though again these increases did not differ substantially from those in the year prior to switch to these INSTIs. Among those underweight or healthy weight at time of switch, 13% (95% CI: 5%, 29%), 18% (95% CI: 11%, 29%), 36% (95% CI: 21%, 54%), and 12% (95% CI: 3%, 34%) of females and 6% (95% CI: 1%, 27%), 8% (95% CI: 3%, 21%), 12% (95% CI: 4%, 29%), and 11% (95% CI: 3%, 31%) of males switching to BIC, DTG, EVG, and RAL were overweight/obese by 2 years after switch, respectively.

## DISCUSSION

In a relatively large cohort of treatment-experienced AYAPHIV in the United States, the majority maintained or achieved viral suppression after switch to an INSTI-based regimen. Though not substantially different from weight gain prior to switch, increases in weight and BMI through 2 years after switch to BIC, DTG, EVG, or RAL were observed at varying levels among females and males.

Despite decreases in average HIV viral loads in the first year after switch to all INSTIs, the proportion of AYAPHIV virally suppressed at <200 copies/mL after switch to an INSTI-based regimen was moderate, with 74% and 69% virally suppressed 1 and 2 years after switch to BIC and 62% and 60% virally suppressed 1 and 2 years after switch to DTG. Though only 43% and 51% of switches to BIC and DTG occurred while viremic, these results are comparable to those observed in trials of INSTIs among viremic treatment–experienced adults [12, 13]. Specifically, the SAILING trial reported viral suppression at <50 copies/mL to be 71% at 48 weeks among viremic treatment-experienced adults who received DTG [12]. Our results are also similar to the IMPAACT 1093 study of 23 young adolescents with detectable HIV viral load, which reported viral suppression at <400 copies/mL to be 74% at 48 weeks after receipt of DTG [38]. For this study, we could not assess reasons for virologic failure such as adherence issues or accumulated resistance during follow-up. This is relevant as most currently available long-acting injectable strategies include cabotegravir, an INSTI whose activity could be compromised with development of resistance on other INSTI-based regimens.

Among our population of treatment-experienced AYAPHIV, we observed increases in weight of about 3 kg in the first year after switch to DTG or EVG, with no substantial differences between females and males. This increase is comparable to what was reported in a pooled analysis of weight gain in 8 randomized controlled clinical trials of treatment-naive adults with HIV, which estimated an increase of 2.3 kg at 48 weeks after initiation of any INSTI and 2.8 kg at 48 weeks after initiation of DTG [24]. Though the study also reported weight gain of 3.1 kg at 48 weeks after initiation of BIC, we did not observe substantial weight gain overall in the 1 year after switch to BIC in our population. While weight gain after switch to particular INSTIs did not substantially differ from weight increases prior to switch, the continued weight gain on INSTI-based regimens is concerning, particularly for AYAPHIV who are also more likely to have been exposed in childhood to thymidine analogues such as stavudine and/or didanosine, which have been associated with visceral fat accumulation [39].

Over a third of our study population was overweight/obese at the time of switch to an INSTI-based regimen and ~15% of those underweight/healthy weight at time of switch were overweight/ obese by 2 years after switch to an INSTI. The proportion over-weight/obese also increased over time in the pooled analysis of weight gain among treatment-naive adults with HIV from 47.7% at baseline to 53.4% at 48 weeks [33]. That study did not observe a significant clinical impact of weight gain on cardiometabolic parameters in their study population due to short duration of follow-up. However, becoming overweight/obese will likely exacerbate existing cardiovascular disease risk among AYAPHIV as they age into adulthood with a life long history of and ongoing exposure to HIV and ART [40]. It will be important to monitor the clinical consequences of weight gain on the long-term cardiometabolic health of AYAPHIV.

While our study population was relatively large, after subsetting by INSTI and sex at birth, our sample size became limited to further stratify by other risk factors for weight gain such as age, use of tenofovir alafenamide (TAF), and race/ethnicity. Weight gain may differ for adolescents and young adults compared to adults due to differences in pubertal and sexual maturation. Prior literature among adults with HIV has also shown larger weight gain with use of TAF and among females who self-report as Black [24]. We were also interested in estimating return-to-health after switch to an INSTI regimen but were limited in the sample size of participants with low CD4 counts within each INSTI and sex at birth analysis subset. We were also unable to stratify our analyses based on ART received prior to switch due to incomplete histories of lifetime ART for AMP Up participants. As this was primarily a descriptive study to quantify HIV viral load, CD4 counts, weight, and BMI after switch to specific INSTIs, further studies are warranted to identify risk factors for weight gain, particularly for those who became overweight/obese after switch to an INSTI.

In summary, this study of a large, diverse cohort of AYAPHIV switching to an INSTI-based regimen shows moderate effectiveness of the newer INSTIs, BIC, and DTG. These data may help providers develop realistic viral suppression targets for this heavily treatment-experienced population. This study also shows variable continued average weight gain after switch to specific INSTIs, which has implications for the long-term cardiometabolic health of this population.

## Figures and Tables

**Table 1.** Characteristics at Time of INSTI-based Regimen Switch

	All^a^ (N = 556)	Bictegravir (N = 167)	Dolutegravir (N = 282)	Elvitegravir (N = 189)	Raltegravir (N = 151)
Age (y)	20.7 (17.4, 24.6)	24.0 (21.2, 26.3)	21.9 (18.9, 26.1)	21.2 (18.4, 24.1)	17.4 (14.6, 21.6)
Female sex at birth	347 (62%)	112 (67%)	195 (69%)	117 (62%)	91 (60%)
Self-reported race/ethnicity					
Black non-Hispanic	339 (61%)	108 (65%)	163 (58%)	136 (72%)	86 (57%)
White non-Hispanic	39 (7%)	12 (7%)	20 (7%)	11 (6%)	8 (5%)
Hispanic (regardless of race)	159 (29%)	43 (26%)	86 (30%)	40 (21%)	53 (35%)
More than one race/other race	15 (3%)	4 (2%)	10 (4%)	2 (1%)	3 (2%)
Missing	4 (1%)	0 (0%)	3 (1%)	0 (0%)	1 (1%)
HIV viral load (log_10_ copies/mL)^b^	2.22 (1.30, 3.76)	1.88 (1.30, 3.37)	2.05 (1.30, 3.71)	1.62 (1.30, 3.19)	3.43 (1.88, 4.29)
Missing	15 (3%)	10 (6%)	8 (3%)	6 (3%)	5 (3%)
CD4 count (cells/mm^3^)^b^	526 (283, 744)	478 (303, 772)	480 (217, 727)	567 (302, 777)	462 (268, 668)
Missing	14 (3%)	7 (4%)	10 (4%)	4 (2%)	4 (3%)
CDC class C clinical stage	157 (28%)	43 (26%)	94 (33%)	50 (26%)	46 (30%)
Prior ARV regimen^c^					
ART with INSTI/EI/FI	12 (2%)	79 (47%)	53 (19%)	31 (16%)	16 (11%)
ART with PI	165 (30%)	17 (10%)	57 (20%)	46 (24%)	57 (38%)
ART with NNRTI	66 (12%)	14 (8%)	22 (8%)	23 (12%)	12 (8%)
Other ARV	28 (5%)	9 (5%)	14 (5%)	5 (3%)	12 (8%)
No ARV	63 (11%)	24 (14%)	27 (10%)	30 (16%)	20 (13%)
Missing	222 (40%)	24 (14%)	109 (39%)	54 (29%)	34 (23%)
INSTI regimen included TAF	194 (35%)	167 (100%)	69 (24%)	115 (61%)	4 (3%)

Summary statistics presented as median (Q1, Q3) or N (%).

Abbreviations: ART, antiretroviral treatment; ARV, antiretroviral; BMI, body mass index; CDC, Centers for Disease Control and Prevention; EI, entry inhibitor; FI, fusion inhibitor; INSTI, integrase strand transfer inhibitor; NNRTI, non-nucleoside reverse transcriptase inhibitor; PI, protease inhibitor; TAF, tenofovir alafenamide fumarate.

a The “All” column represents the first time a participant was on any specific INSTI regimen, whereas the 4 specific INSTI columns represent the time that participants first initiated those specific INSTI regimens; therefore, the “All” column does not represent a total of the subsequent 4 columns.

b Nearest measure up to +/−14 d for HIV viral load and +/−30 d for CD4, weight, and BMI. If no measure available within these windows, linear interpolation was performed using the closest available pre- and post-INSTI initiation measures.

c Prior ARV regimen was generally unavailable for participants who enrolled in AMP Up already on an INSTI-based regimen.

**Table 2.** Characteristics at Time of INSTI-based Regimen Discontinuation

	Did Not Discontinue^a^ (N = 428)	Bictegravir (N = 26)	Dolutegravir (N = 127)	Elvitegravir (N = 99)	Raltegravir (N = 109)
Age (y)	26.5 (24.0, 30.5)	26.0 (23.5, 30.7)	23.9 (20.7, 27.6)	22.5 (20.9, 25.7)	20.1 (16.6, 24.2)
Female sex at birth	264 (62%)	21 (81%)	95 (75%)	70 (71%)	65 (60%)
Pregnancy within prior 40 wks	1 (0%)	2 (10%)	5 (5%)	11 (16%)	7 (11%)
Pregnancy within prior 13 wks	0 (0%)	0 (0%)	4 (4%)	7 (10%)	1 (2%)
Self-reported race/ethnicity					
Black non-Hispanic	254 (59%)	17 (65%)	79 (62%)	73 (74%)	70 (64%)
White non-Hispanic	31 (7%)	1 (4%)	9 (7%)	5 (5%)	5 (5%)
Hispanic (regardless of race)	128 (30%)	7 (27%)	34 (27%)	21 (21%)	32 (29%)
More than one race/other race	13 (3)	1 (4%)	3 (2%)	0 (0%)	2 (2%)
Missing	2 (0%)	0 (0%)	2 (2%)	0 (0%)	0 (0%)
HIV viral load (log_10_ copies/mL)^b^	1.5 (1.3, 2.4)	2.7 (1.3, 3.7)	2.2 (1.3, 3.8)	2.5 (1.5, 3.9)	2.6 (1.6, 4.1)
Missing	246 (57%)	3 (12%)	6 (5%)	5 (5%)	4 (4%)
CD4 count (cells/mm^3^)^b^	558 (364, 746)	464 (242, 677)	427 (229, 646)	447 (255, 725)	455 (164, 686)
Missing	247 (58%)	1 (4%)	7 (6%)	4 (4%)	4 (4%)
Weight (kg)^b^	66.1 (57.6, 80.3)	64.9 (54.1, 75.4)	61.2 (54.9, 73.3)	67.3 (56.3, 78.3)	59.1 (51.7, 78.2)
Missing	193 (45%)	4 (15%)	20 (16%)	10 (10%)	9 (8%)
BMI (kg/m^2^)^b^					
Underweight (<18.5)	10 (2%)	2 (8%)	7 (6%)	3 (3%)	9 (8%)
Healthy weight (18.5 to <25)	99 (23%)	9 (35%)	55 (43%)	38 (38%)	49 (45%)
Overweight (25.0 to <30)	55 (13%)	6 (23%)	20 (16%)	20 (20%)	19 (17%)
Obese (≥30)	42 (10%)	4 (15%)	21 (17%)	22 (22%)	18 (17%)
Missing	222 (52%)	5 (19%)	24 (19%)	16 (16%)	14 (13%)

Summary statistics presented as Median (Q1, Q3) or N (%). For pregnancy, denominator only included females for calculation of %.

Abbreviations: BMI, body mass index; INSTI, integrase strand transfer inhibitor.

a Characteristics at end of follow-up instead of at time of INSTI-regimen discontinuation.

b Nearest measure up to −90/+7 d for VL and −90/+30 d for CD4, weight, and BMI. If no measure available within these windows, linear interpolation was performed using the closest available pre- and post-INSTI discontinuation measures.
